# Responses to textual and pictorial cigarette pack health warnings: findings from an exploratory cross-sectional survey study in Austria

**DOI:** 10.1186/s12889-018-5342-8

**Published:** 2018-04-03

**Authors:** Hannes Mayerl, Erwin Stolz, Wolfgang Freidl

**Affiliations:** Institute of Social Medicine and Epidemiology, Medical University of Graz, Universitaetsstrasse 6/I, Graz, 8010 Austria

**Keywords:** smoking, cigarette packs, health warnings, packaging and labelling

## Abstract

**Background:**

Packaging and labelling tobacco products has emerged as an effective policy to reduce the global health burden of tobacco use. The main purpose of this study was to explore Austrian smokers’ and ex-smokers’ responses to both the textual and pictorial cigarette pack health warnings (CPHWs) recently implemented.

**Methods:**

We analysed self-reported data (*N*=500) from an Austrian cross-sectional survey that was conducted after the implementation of the new pictorial CPHWs in 2016.

**Results:**

The results showed only weak effects of the CPHWs on the decision to quit or reduce smoking, and the level of impact of the CPHWs remained limited particularly because of smokers denying the ill-effects of tobacco use.

**Conclusions:**

Although the CPHWs seem to have the potential to promote a change in smoking behaviour, the warnings reached only a rather small group of smokers, while the majority of smokers appeared to remain unaffected by this intervention. Public health policies are challenged to increase the salience of CPHWs and to overcome smokers’ denial of detrimental health effects.

## Background

Despite recent declines in smoking prevalence in a majority of countries worldwide [1], smoking still represents a major public health concern. The World Health Organisation (WHO) estimated the global proportion of all deaths attributable to tobacco use to be 12 % among adults aged 30 years or over [2].

In recognition of the global health burden of tobacco use, the WHO *Framework Convention on Tobacco Control (FCTC)* has been adopted, prompting the participating nations to implement a set of tobacco control policies. One of these policies concerns packaging and labelling: each tobacco product must carry clear, visible and legible health warnings on the package (Article 11) [3]. This policy has emerged as a cost-effective intervention when it comes to communicating the health risks of smoking to a broad audience of smokers [4].

To date, 181 countries are parties to the FCTC, and the Austrian government ratified this convention in 2005. In accordance with the FCTC and with European guidelines (see Tobacco Products Directive 2014/40/EU) [5], the revised version of the Austrian Tobacco Act [6] requires each tobacco product to carry (1) a general textual warning (“Smoking kills – quit now.”), (2) a general message (“Tobacco smoke contains more than 70 substances that have been proved to be carcinogenic.”), and (3) a combination of an additional textual warning (e.g., “Smoking damages your lungs.”) with a matching picture showing the health effects caused by smoking. These cigarette pack health warnings (CPHWs) are required to cover 65% of each the front and rear side of the tobacco product.

A number of smokers’ responses to CPHWs have been documented in previous studies, and pictorial warnings have been found to be more effective in terms of provoking cognitive, emotional, and behavioural reactions than text-only warnings [4, 7–10]. In general, CPHWs have been shown to generate knowledge and reflection about the health risks of smoking [7, 10, 11]; to reduce the odds of starting to smoke in adolescence [12]; and to increase the likelihood of avoiding cigarettes, making attempts to quit, or actually quitting smoking [11, 13, 14].

Comparable data for Austria after implementation of the new CPHWs in 2016 are however lacking. The main objective of this study thus was to explore smokers’ responses to both textual and pictorial CPHWs and to analyse how these responses were related to intentions to quit smoking.

## Methods

### Study design and data

The present study is based on survey data concerning smoking behaviour and responses to CPHWs. Data was collected from February to March 2017 by *The Institute for Empirical Social Studies (IFES)* on behalf of the authors. A random sample of 500 persons was drawn from the Austrian population of both current smokers and ex-smokers (i.e. smoking cessation during the last 12 months). Given that smoking usually develops in the period from adolescence to young adulthood [15] and that an early onset of smoking is strongly related to future smoking patterns [16, 17], smoking prevention is of great public health interest among the younger age group in particular. We therefore aimed for a disproportionately higher rate of 15–23 year olds in order to increase statistical power to detect potential differences between the younger and the older age groups. Self-reported data were collected using computer-assisted telephone and web interviews (CATI/CAWI).

### Measures

The conceptualization of the survey variables was largely based on previous survey studies examining the effects of CPHWs on smokers’ behaviour and intentions [7, 18–21]. The variables used in this study comprised behavioural, cognitive, and emotional responses to the CPHWs, measures of salience of the CPHWs, smokers’ assessments of the health risks of smoking, smokers’ intentions to quit, smoking history and dependence, and socio-economic and socio-demographic factors.

#### Smoking status

Participants were categorized according to their smoking status, i.e. to the fact whether they smoked every day (*regular smokers*) or occasionally (*occasional smokers*), or whether they were ex-smokers that had quit smoking during the last 12 months (*ex-smokers*).

#### Smoking cessation because of pictorial CPHWs

To assess the direct effects of health warnings on the decision to quit smoking, ex-smokers were asked to declare whether or not the new cigarette packs with the pictorial warnings were the main reason or at least one of the reasons why they quit smoking. Response options were: *0 =no*; *1 =yes, one reason among others*; *2 =yes, the main reason*.

#### Intention to quit smoking

To quantify quitting intentions, regular and occasional smokers (but not ex-smokers) were asked how strongly they wanted to quit smoking. Response categories were: *0 =not at all*; *1 =somewhat*; *2 =quite a lot*; *3 =a lot*. In order to facilitate interpretability in subsequent regression analysis, we dichotomized this variable by combining response categories 1, 2, and 3. The resulting categories thereby represented no intention to quit smoking on the one hand, and at least a slight intention to quit smoking on the other hand.

#### Warnings impact score (WIS)

In order to obtain a total score for the impact of textual and pictorial health warnings, we summed up the dichotomized scores of four items (for a similar approach see [20]):

1. Change in feelings because of the new CPHWs: Regular and occasional smokers were asked whether or not the new CPHWs had changed their feelings about smoking. Response options were: *0 =no, feelings have not changed*; *1 =yes, I feel bad smoking*; *2 =yes, I feel very bad smoking*. We dichotomized this item score by combining response categories 1 and 2 into one category.
2. Forgoing cigarettes because of pictorial CPHWs: Regular and occasional smokers were asked to specify whether or not they had reduced cigarette consumption due to the pictures on the packs. Response categories were: *0 =no*; *1 =yes, I smoke a little less*; *1 =yes, I smoke much less*. Again, we combined response categories 1 and 2 to dichotomize this item score.
3. Frequency of reading warnings: Regular and occasional smokers were requested to indicate how often they had read the textual warnings on the cigarette packs during the last weeks. Response categories were: *0 =never*; *1 =once*; *2 =repeatedly*; *3 =often*; *4 =almost always*. For dichotomization, we combined response categories 0–1 and 2–4 into one category, respectively.
4. General attitude toward pictorial warnings: Regular and occasional smokers were asked whether or not they would agree that it is generally a good idea to show pictorial warnings on the cigarette packs as a deterrent (*0 =no*; *1 =yes*).

Higher values on the WIS represented more positive responses in terms of provoking the effects intended by the health warnings. This sum score was then used in regression analysis. Confirmatory factor analysis confirmed the one-dimensional structure of this scale, and internal consistency was sufficient (Cronbach’s *α*=0.75).

#### Assessment of the validity of warnings

Regular and occasional smokers were requested to state whether they believed that the warnings on the cigarette packs are valid, on a 7-point rating scale ranging from *1 =very untrue of what I believe*, to *7 =very true of what I believe*.

#### Smoking risk assessment

To quantify smokers’ knowledge about the health risks of smoking, regular and occasional smokers were requested to assess (1) smokers’ risk (compared to non-smokers of the same age and sex) to develop a life-threatening disease, (2) smokers’ risk to develop a life-threatening disease while they keep smoking, and (3) smokers’ risk to die earlier than if they quit smoking. Response categories ranged from *1 =my risk is much lower* to *7 =my risk is much higher* for the first item and from *1 =very low* to *7 =very high* for the latter two items, on a 7-point Likert-scale, respectively. We summed up the three item scores and used the resulting sum score in a subsequent analysis. Internal consistency was found to be good (Cronbach’s *α*=0.86).

#### Use of neutral covers

Regular and occasional smokers were asked to indicate whether or not they used a neutral cover to hide the pictures on the cigarette packs (*0 =no*; *1 =yes*).

#### Cigarette dependence

The measure for cigarette dependence was based on the score used in the *Fagerström Test for Cigarette Dependence (FTCD)* [22, 23]. Internal consistency was found to be good in the present study (Cronbach’s *α*=0.82). Again, these test items were only presented to regular and occasional smokers.

#### Additional variables

Both the responses to the CPHWs and the intentions to quit smoking may be affected by individual-level differences in factors such as smoking history and socio-demographic/socio-economic backgrounds [10, 20, 24, 25]. We thus considered the number of years smoking (≤3 years; 4–10 years; >10 years), household income (≤2000 Euros; 2001–3000 Euros; >3000 Euros), educational level (highest level reached: still in school or compulsory education; apprenticeship certificate or diploma from vocational school; high school diploma; university degree); age group (15–23 years vs. >23 years), and sex (male vs. female).

### Statistical analysis

First, descriptive analyses were used to explore the sample characteristics for both regular and occasional smokers, each stratified by age group. We conducted *χ*^2^-tests of independence and *t*-tests in order to investigate potential differences with regard to smoking status and age group. Second, we used multiple linear regression analysis in order to examine the determinants of the health warnings impact (WIS) and multiple logistic regression analysis in order to predict the intention to quit smoking using the WIS. Other predictor variables included the assessment of the validity of warnings, smoking risk assessment, the question whether or not neutral covers were used, the cigarette dependence score (FTCD), smoking status, and the additional variables.

In total, 5.08 % of all data used in this study were missing. The proportion of missing values across the variables varied between 0 % and 29 %. In order to handle missing data, we repeated regression analysis for each of the *m*=50 multiple imputed datasets and pooled the results according to Rubin’s rules [26]. In the imputation model, we used the entire set of predictor and outcome variables to impute the missing data. All statistical analyses were carried out using R version 3.4.2 [27], and we used the R-package mice version 2.30 for multiple imputation by chained equations [28].

## Results

### Sample characteristics and bivariate analysis

Within the total sample, 331 individuals smoked regularly, 129 individuals smoked occasionally, and 40 individuals had quit smoking in the last 12 months (ex-smokers). 50 % of participants were female, and age was between 16 and 89 years (*M*=37.5, *SD*=15.5). Sample characteristics for all smokers in total and for each group of regular and occasional smokers, stratified by age group, are listed in Table 1.

**Table 1:** Descriptive statistics for all smokers in total and for regular and occasional smokers, stratified by age group

	*M*(*SD*) or *n*(%)				
		15–23 years		>23 years	
	All smokers	Regular	Occasional	Regular	Occasional
*N*	460	55	47	276	82
Intention to quit smoking					
Not at all	123 (27.2)	13 (24.1)	8 (17.4)	83 (30.2)	19 (24.7)
Somewhat	166 (36.7)	20 (37.0)	16 (34.8)	103 (37.5)	27 (35.1)
Quite a lot	100 (22.1)	16 (29.6)	14 (30.4)	52 (18.9)	18 (23.4)
A lot	63 (13.9)	5 (9.3)	8 (17.4)	37 (13.5)	13 (16.9)
Change in feelings because of the new CPHWs					
No, feelings have not changed	381 (84.3)	48 (88.9)	33 (71.7)	239 (87.9)	61 (76.2)
Yes, I feel bad smoking	51 (11.3)	4 (7.4)	10 (21.7)	23 (8.5)	14 (17.5)
Yes, I feel very bad smoking	20 (4.4)	2 (3.7)	3 (6.5)	10 (3.7)	5 (6.2)
Forgoing cigarettes because of pictorial CPHWs					
No	417 (91.4)	46 (85.2)	40 (85.1)	264 (96.0)	67 (83.8)
Yes, I smoke a little less	32 (7.0)	7 (13.0)	5 (10.6)	10 (3.6)	10 (12.5)
Yes, I smoke much less	7 (1.5)	1 (1.9)	2 (4.3)	1 (0.4)	3 (3.8)
Frequency of reading warnings					
Never	182 (39.9)	20 (37.0)	17 (36.2)	114 (41.6)	31 (38.3)
Once	74 (16.2)	14 (25.9)	9 (19.1)	42 (15.3)	9 (11.1)
Repeatedly	103 (22.6)	8 (14.8)	14 (29.8)	60 (21.9)	21 (25.9)
Often	46 (10.1)	4 (7.4)	5 (10.6)	26 (9.5)	11 (13.6)
Almost always	51 (11.2)	8 (14.8)	2 (4.3)	32 (11.7)	9 (11.1)
Attitude toward pictorial warnings (good idea)	151 (34.4)	16 (30.2)	27 (64.3)	67 (25.4)	41 (51.2)
Warnings Impact Score (range: 0–4)	1.0 (1.1)	0.9 (0.9)	1.6 (1.2)	0.8 (1.0)	1.4 (1.1)
Assessment of the validity of warnings (range: 1–7)	4.6 (2.0)	4.1 (2.1)	5.0 (1.7)	4.6 (2.0)	4.8 (2.1)
Smoking risk assessment (range: 3–21)	12.7 (4.1)	12.8 (4.4)	12.9 (4.0)	12.5 (4.1)	13.0 (4.2)
Use of neutral covers (yes)	82 (18.3)	9 (17.0)	6 (13.0)	62 (23.0)	5 (6.2)
Cigarette dependence (FTCD; range: 0–10)	3.2 (2.4)	3.0 (2.0)	0.6 (0.8)	3.8 (2.3)	0.5 (0.8)
Number of years smoking					
≤3 years	69 (15.4)	20 (36.4)	30 (68.2)	10 (3.6)	9 (12.2)
4–10 years	91 (20.3)	35 (63.6)	13 (29.5)	29 (10.5)	14 (18.9)
>10 years	289 (64.4)	0 (0.0)	1 (2.3)	237 (85.9)	51 (68.9)
Household income					
≤2000 Euros	135 (37.3)	22 (52.4)	9 (24.3)	82 (37.8)	22 (33.3)
2001–3000 Euros	141 (39.0)	12 (28.6)	14 (37.8)	81 (37.3)	34 (51.5)
>3000 Euros	86 (23.8)	8 (19.0)	14 (37.8)	54 (24.9)	10 (15.2)
Educational level					
Still in school/compulsory education	54 (11.7)	12 (21.8)	11 (23.4)	25 (9.1)	6 (7.3)
Apprenticeship certificate/vocational school	210 (45.7)	19 (34.5)	12 (25.5)	153 (55.4)	26 (31.7)
High school diploma	130 (28.3)	23 (41.8)	22 (46.8)	58 (21.0)	27 (32.9)
University degree	66 (14.3)	1 (1.8)	2 (4.3)	40 (14.5)	23 (28.0)
Age (years)	37.4 (15.4)	20.1 (2.0)	20.5 (2.0)	44.0 (14.4)	36.5 (11.1)
Sex (female)	227 (49.3)	32 (58.2)	30 (63.8)	126 (45.7)	39 (47.6)

This table includes data for current smokers but not for ex-smokers because most of these questions concern current smoking behaviour and current response to the CPHWs. The relevant questions were thus only addressed to smokers. We report the mean (*M*) and the standard deviation (*SD*) for continuous measures, and the absolute frequencies (*n*) and percentages (%) for categorical measures. *FTCD*, Fagerström Test for Cigarette Dependence

#### Smoking cessation because of pictorial CPHWs

None of the ex-smokers declared that the pictorial warnings on the cigarette packs were the main reason for smoking cessation. Only 4 persons (10.0 %) indicated that the pictures were among the reasons for quitting, and 36 persons (90.0 %) denied that the pictorial warnings had any effect on the decision to quit smoking.

#### Intention to quit smoking

27.2 % of the smokers denied any intention to quit smoking, while the remaining portion indicated at least a slight intention to quit. No significant difference for smoking status (*χ*^2^(1)=2.1, *p*=0.156) and age group (*χ*^2^(1)=2.1, *p*=0.146) was found.

#### Change in feelings because of the new CPHWs

84.3 % of the smokers reported no change in feelings because of the new packs, while a percentage of 15.7 % indicated that they now felt bad or very bad smoking. In this regard, occasional smokers (25.4 %) were found to be more likely to experience bad or very bad feelings than regular smokers (12.0 %; *χ*^2^(1)=11.4, *p*<0.001). Age had no effect (*χ*^2^(1)=0.76, *p*=0.385).

#### Foregoing cigarettes because of pictorial CPHWs

A vast majority of smokers (91.5 %) indicated that they had not reduced cigarette consumption due to the pictorial warnings, while the remaining portion reduced smoking at least a little. A higher proportion of occasional smokers (15.7 %), compared to regular smokers (5.8 %), and of the younger age group (14.9 %), compared to the older age group (6.8 %), reported to have reduced their cigarette consumption (*χ*^2^(1)=10.4, *p*=0.001 and *χ*^2^(1)=5.6, *p*=0.018, respectively).

#### Frequency of reading warnings

39.9 % of smokers had never read the warnings on the cigarette packs during the last weeks, while a percentage of 16.2 % had read the warnings once and 43.9 % had read the warnings more than once. No associations were found with regard to smoking status (*χ*^2^(1)=1.3, *p*=0.260) and age group (*χ*^2^(1)=0.4, *p*=0.525).

#### General attitude toward pictorial warnings

Overall, 34.0 % of smokers agreed when asked whether it is generally a good idea to show pictorial warnings on the cigarette packs as a deterrent, and this proportion was lower among regular (26.2 %) than among occasional smokers (55.7 %; *χ*^2^(1)=32.8, *p*<0.001), and lower among smokers aged 24 years or over (31.4 %) than among the younger age group (45.3 %; *χ*^2^(1)=32.8, *p*=0.017).

#### Assessment of the validity of warnings

The assessment with regard to the validity of the warnings did not statistically differ between regular and occasional smokers (*t*(446)=−1.9, *p*=0.063) or between the younger and the older age group (*t*(446)=−0.4, *p*=0.670).

#### Smoking risk assessment

Smoking status and age group also showed no effect on smoking risk assessment (*t*(425)=−0.9, *p*=0.363 and *t*(425)=0.4, *p*=0.669, respectively).

#### Use of neutral covers

A percentage of 18.3 % of smokers reported using neutral covers to hide the pictures on the cigarette packs, and regular smokers (22.0 %) were more likely to use neutral covers than occasional smokers (8.7 %; *χ*^2^(1)=9.8, *p*=0.002). No difference for age group was found (*χ*^2^(1)=0.6, *p*=0.447).

### Determinants of the health warnings impact

Table 2 shows the pooled results of linear regression analysis regarding the impact of the health warnings (WIS). We found significant effects for validity assessment, for smoking risk assessment, cigarette dependence, and smoking status. An increasing endorsement of the validity of CPHWs and increasing levels of smoking risk assessment were related to higher values on WIS. The confidence intervals of the standardized coefficients also suggested that the effects of these two predictor variables did not differ considerably. Increasing cigarette dependence and regular (vs. occasional) smoking status, in turn, were related to lower levels on WIS.

**Table 2:** Linear regression analysis for the Warnings Impact Score (WIS)

	Warnings impact score		
	*b*	*β* [95% CI]	*p*
(Intercept)	−0.81	0.03 [ −0.33, 0.40]	0.86
Assessment of the validity of warnings	0.06	0.13 [0.03, 0.22]	0.01
Smoking risk assessment	0.05	0.22 [0.13, 0.32]	0.00
Use of neutral covers (ref.: no)			
Yes	−0.01	−0.01 [ −0.24, 0.22]	0.96
Cigarette dependence (score FTCD)	−0.05	−0.13 [ −0.25, −0.01]	0.04
Smoking status (ref.: regularly)			
Occasionally	0.29	0.29 [0.04, 0.54]	0.02
Number of years smoking (ref.: ≤3 years)			
4–10 years	−0.09	−0.09 [ −0.40, 0.23]	0.59
>10 years	−0.35	−0.35 [ −0.70, 0.00]	0.05
Household income (ref.: ≤2000 Euros)			
2001–3000 Euros	0.03	0.03 [ −0.19, 0.25]	0.82
>3000 Euros	0.04	0.04 [ −0.22, 0.30]	0.76
Educational level (ref.: still in school/compulsory)			
Apprenticeship/vocational	−0.13	−0.13 [ −0.43, 0.16]	0.38
High school diploma	0.15	0.15 [ −0.16, 0.46]	0.36
University degree	−0.11	−0.11 [ −0.49, 0.26]	0.55
Age group (ref.: 15–23 years)			
>23 years	0.19	0.19 [ −0.13, 0.50]	0.25
Sex (ref.: male)			
Female	−0.01	−0.01 [ −0.19, 0.17]	0.95

*N*=460. Pooled *R*^2^=0.204. *ref.*, reference group. *b*, unstandardized coefficient. *β*, standardized coefficient. *CI*, Confidence interval. *p*, *p*-value. *FTCD*, Fagerström Test for Cigarette Dependence

### Predicting the intention to quit smoking

Table 3 shows the coefficients from the logistic regression analysis predicting the intention to quit smoking. We found two significant effects: higher values on WIS as well as increased smoking risk assessment were related to increased odds to report at least a slight intention to quit. The odds ratios also suggested a largely identical effect size for these two predictor variables.

**Table 3:** Logistic regression analysis for the intention to quit smoking

	Intention to quit smoking (ref.: not at all)		
	*b*	OR [95% CI]	*p*
(Intercept)	0.66	1.93 [0.74, 5.01]	0.18
Warnings Impact Score	0.60	1.82 [1.32, 2.51]	0.00
Assessment of the validity of warnings	−0.06	0.94 [0.74, 1.20]	0.64
Smoking risk assessment	0.54	1.72 [1.29, 2.28]	0.00
Use of neutral covers (ref.: no)			
Yes	−0.29	0.75 [0.42, 1.34]	0.33
Cigarette dependence (FTCD)	0.05	1.05 [0.78, 1.42]	0.74
Smoking status (ref.: regularly)			
Occasionally	0.06	1.06 [0.54, 2.08]	0.87
Number of years smoking (ref.: ≤3 years)			
4–10 years	0.73	2.07 [0.84, 5.07]	0.11
>10 years	−0.09	0.91 [0.35, 2.40]	0.86
Household income (ref.: ≤2000 Euros)			
2001–3000 Euros	−0.10	0.90 [0.51, 1.60]	0.72
>3000 Euros	0.03	1.03 [0.52, 2.03]	0.93
Educational level (ref.: still in school/compulsory)			
Apprenticeship/vocational	0.61	1.84 [0.89, 3.79]	0.10
High school diploma	0.37	1.45 [0.68, 3.12]	0.34
University degree	0.47	1.60 [0.63, 4.07]	0.32
Age group (ref.: 15–23 years)			
>23 years	−0.04	0.96 [0.38, 2.43]	0.93
Sex (ref.: male)			
Female	0.18	1.20 [0.75, 1.92]	0.45

*N*=460. Pooled pseudo- *R*^2^ [36–38]: McFadden, 0.122; Cox & Snell, 0.133; Nagelkerke, 0.192. *WIS*, Warnings Impact Score. *ref.*, Reference group. *b*, unstandardised regression coefficient. *OR*, odds ratio. *CI*, Confidence interval. *p*, *p*-value. *FTCD*, Fagerström Test for Cigarette Dependence. Continuous variables were included as standardized measures

## Discussion

This paper constitutes the first study exploring and analysing smokers’ and ex-smokers’ responses to the newly implemented CPHWs in Austria. Although we found that the new packaging and labelling has a potential to persuade smokers to reduce or quit smoking, these warnings affected only a rather small group of smokers, while the vast majority appeared to remain rather unimpressed by this intervention. More specifically, 90 % of the ex-smokers denied any effect of the pictorial warnings on their decision to quit smoking and a similar proportion of smokers had not noticed any reduction in their cigarette consumption because of the pictures. These findings are comparable to a previous study that found slightly more than one tenth of European smokers to report cutting down on cigarettes due to health warnings, and this proportion was found to be higher in countries where pictorial warnings were already implemented (vs. countries with text-only warnings) [29].

The reasons for the rather weak impact of the warnings are diverse. One of the reasons concerns the fact that the general willingness to change smoking behaviour was quite weak in the sample. Arguably, it is much harder to convince poorly motivated individuals to stop or reduce smoking than highly motivated individuals (see e.g. [30]), particularly when using simple interventions that can be easily circumvented by consumers if they wish. In fact, more than half of the smokers had read the warnings on the cigarette packs at most once in the last weeks and a considerable proportion also indicated using neutral covers to hide the warnings on the packs. Such avoidance strategies likely prevent the warnings from taking effect. A longitudinal study has shown smokers who more strongly dealt with the warnings (reading, reflecting, discussing them) were more likely to have quit or reduced smoking three months later [31].

A further reason may reside in negative attitudes toward and beliefs about CPHWs in general. Only about one quarter of the regular smokers thought that the new cigarette packs were a reasonable intervention and a considerable portion of smokers even doubted the validity of the health warnings. The latter finding may be a result of smokers’ attempts to reduce cognitive conflict between the desire to continue smoking and its well-known detrimental health effects [32]. Doubting the validity of health-related information would be one of the strategies to eliminate or reduce such conflict. McMaster and Lee [33] showed that smokers (vs. non- and ex-smokers) estimated their own risk for developing lung cancer to be less than that of an “average smoker”, and smokers more often endorsed obviously untrue statements to justify smoking, even though there was no difference in factual knowledge about the risks of tobacco use between smokers and non- or ex-smokers.

In our study, we also found that the impact of the health warnings was less strong for smokers who doubted the validity of the warnings and the risks of smoking. Displaying the CPHWs – whether textual or pictorial – is probably not enough to overcome smokers’ denial and, eventually motivate them to consider a change in smoking behaviour [33]. Future studies should further examine the mechanisms that underlie the link between CPHWs and smoking behaviour, in order to identify ways of improving the efficacy of the health warnings. A recent meta-analysis of experimental studies demonstrated that there exists an array of psychosocial constructs potentially mediating the relationship between the warnings and changes in intentions and smoking behaviour [9]. Besides cognitive (e.g. denial of health risks), emotional (e.g. fear as a response to the warnings), and behavioural (e.g. avoiding the warnings) factors, the authors of the meta-analysis also recommended to analyse how social factors (e.g. social interactions with peers) influence effectiveness of the warnings.

### Strengths and limitations

The present study explores smokers’ and ex-smokers’ responses to the CPHWs newly implemented in Austria in 2016. This research thus deals with a topical public health issue and our findings might be of interest for public health policies aiming to reduce the global burden of tobacco use. The comprehensive questionnaire used in this survey study allowed us to provide information on different facets of smoking-related issues (e.g. cognitive, affective, and behavioural responses to the CPHWs) on the one hand and to reveal potential factors that may limit the level of impact of the CPHWs on the other hand. Finally, the scales used in this study showed satisfactory psychometric properties in terms of internal consistency and dimensionality.

One shortcoming of the current study resides in the cross-sectional nature of the survey design. The revealed relationships are only correlational and do not permit inferences about cause and effect. A second limitation concerns the self-report character of the analysed data. It is possible that the health warnings also operate on a subconscious or implicit level (see e.g. [34]) and that smokers/ex-smokers may thereby fail to explicitly notice potential effects of the warnings. And thirdly, the relatively small sample sizes in some of the subgroups (i.e. the ex-smokers) compromises generalizability of the findings for these subgroups.

## Conclusions

In conclusion, although we found that the CPHWs might have a potential to promote a change in smoking behaviour, the impact of the warnings seemed to be less strong among “heavy” smokers and among smokers who questioned the detrimental effects smoking can have on their health. The main challenges for public health policies will thus be to increase the salience of health warnings in order to enhance the cognitive processing of smoking-related information and to overcome smokers’ denial of the well-publicised ill-effects of tobacco use [9, 31, 35].

## Abbreviations

CPHWs: Cigarette pack health warnings
FCTC: Framework convention on tobacco control
FTCD: Fagerström test for cigarette dependence
IFES: The Institute for empirical social studies
WHO: World Health Organisation
WIS: Warnings impact score
